# Trends in Tuberculosis — United States, 2013

**Published:** 2014-03-21

**Authors:** Negar Niki Alami, Courtney M. Yuen, Roque Miramontes, Robert Pratt, Sandy F. Price, Thomas R. Navin

**Affiliations:** 1 EIS officer, CDC; 2 Division of TB Elimination, National Center for HIV/AIDS, Viral Hepatitis, STD, and TB Prevention, CDC

In 2013, a total of 9,588 new tuberculosis (TB) cases were reported in the United States, with an incidence rate of 3.0 cases per 100,000 population, a decrease of 4.2% from 2012 (1). This report summarizes provisional TB surveillance data reported to CDC in 2013. Although case counts and incidence rates continue to decline, certain populations are disproportionately affected. The TB incidence rate among foreign-born persons in 2013 was approximately 13 times greater than the incidence rate among U.S.-born persons, and the proportion of TB cases occurring in foreign-born persons continues to increase, reaching 64.6% in 2013. Racial/ethnic disparities in TB incidence persist, with TB rates among non-Hispanic Asians almost 26 times greater than among non-Hispanic whites. Four states (California, Texas, New York, and Florida), home to approximately one third of the U.S. population, accounted for approximately half the TB cases reported in 2013. The proportion of TB cases occurring in these four states increased from 49.9% in 2012 to 51.3% in 2013. Continued progress toward TB elimination in the United States will require focused TB control efforts among populations and in geographic areas with disproportionate burdens of TB.

Health departments in the 50 states and the District of Columbia electronically report to CDC verified cases of disease that meet the CDC and Council of State and Territorial Epidemiologists surveillance case definition for TB.* Reports include the patient’s country of origin, self-identified race and ethnicity (i.e., Hispanic or non-Hispanic), information on risk factors (e.g., homelessness and incarceration), human immunodeficiency virus (HIV) status, and drug-susceptibility test results. CDC calculates national and state TB incidence rates overall and by racial/ethnic group, using U.S. Census Bureau population estimates (2). The Current Population Survey provides the population denominators used to calculate TB incidence rates and percentage changes according to national origin.† For TB surveillance, a U.S.-born person is defined as a person born in the United States or its associated jurisdictions,§ or a person born in a foreign country but having at least one U.S.-citizen parent. In 2013, the country of birth was unknown for 0.4% of patients, and race/ethnicity was unknown for 0.4%. In this report, persons of Hispanic ethnicity might be of any race; non-Hispanic persons are categorized as Asian, black, white, American Indian/Alaska Native, Native Hawaiian or other Pacific Islander, or of multiple races.

Compared with the national TB incidence rate of 3.0 cases per 100,000 population, the median incidence rate in reporting areas was 2.2 per 100,000 population, ranging from zero in Wyoming to 9.7 per 100,000 population in Alaska (Figure 1). Thirty-three states had lower rates in 2013 than in 2012. Nine states and the District of Columbia had incidence rates higher than the national rate. In 2013, as in 2012, four states (California, Texas, New York, and Florida) reported more than 500 cases each. Combined, these four states accounted for 4,917 TB cases, 51.3% of all TB cases reported in 2013.

Among U.S.-born persons, the number and rate of TB cases decreased in 2013. The 3,377 TB cases reported among U.S.-born persons (35.4% of all cases with known national origin) were 7.6% fewer than the number reported in 2012 and 61.0% fewer than the number reported in 2000 (Figure 2). The rate of 1.2 per 100,000 population among U.S.-born persons is an 8.4% decrease since 2012 and a 64.7% decrease since 2000.

Among foreign-born persons in the United States, the number and rate of TB cases also decreased in 2013. A total of 6,172 TB cases were reported among foreign-born persons (64.6% of all cases in persons with known national origin), a 1.6% decrease since 2012 and a 19.0% decrease since 2000. The 15.6 cases per 100,000 population TB rate among foreign-born persons is a 2.1% decrease since 2012 and a 41.1% decrease since 2000. In 2013, 54.2% of foreign-born persons with TB and known country of birth originated from five countries: 1,233 (20.0%) from Mexico, 776 (12.6%) from the Philippines, 495 (8.0%) from India, 454 (7.4%) from Vietnam, and 377 (6.1%) from China.

The TB incidence rate among Asians was the highest among all racial/ethnic groups and was 25.9 times higher than the incidence rate among whites (Table). Although incidence rates among all racial/ethnic groups declined in 2013, the decrease was greater among whites (9.2%) and blacks (7.5%) than among Hispanics (5.3%) and Asians (0.3%). Among persons with TB, 95% of Asians, 75% of Hispanics, 40% of blacks, and 23% of whites were foreign-born. Among U.S.-born persons, the incidence rate among blacks was 6.2 times higher than among whites.

HIV status was known for 85% of TB cases reported in 2013, as in 2012. Among TB patients with known HIV status, 6.8% had a positive test result for HIV infection in 2013, compared with 7.4% in 2012.

Among persons aged ≥15 years with TB, 98.5% had known housing status, 5.7% of whom reported being homeless within the past year. Among persons aged ≥15 years with TB, 99.1% had a known incarceration status, 3.9% of whom were confined to a detention or correctional facility at the time of TB diagnosis.

A total of 86 cases of multidrug-resistant TB (MDR TB)¶ were reported in 2012, the most recent year for which complete drug-susceptibility results are available. Drug-susceptibility test results for isoniazid and rifampin were reported for 97.9% and 97.6% of cases with culture results positive for *Mycobacterium tuberculosis* in 2011 and 2012, respectively. Among these cases, the percentage of MDR TB for 2012 (1.2% [86 of 7,426 cases]) decreased from the percentage in 2011 (1.6% [129 of 7,906 cases]). The percentage of MDR TB cases among persons without a previous history of TB (1.0%) and the percentage of MDR TB cases among persons with a previous history of TB (3.4%) were lower in 2012 than in 2011. Foreign-born persons accounted for 88.4% of MDR TB cases in 2012. Two cases of extensively drug-resistant TB** have been reported so far for 2013, compared with two cases in 2012 and five cases in 2011.

## Discussion

Despite the continued decline in U.S. TB cases and rates since 1993, the goal of TB elimination in the United States (i.e., less than one case per 1,000,000) set in 1989 (3) remains unmet. Most states reported fewer cases of TB in 2013. However, elevated rates of TB in specific populations remain a major challenge that impedes progress toward TB elimination.

In 2013, four states (California, Texas, New York, and Florida) reported approximately half of the TB cases in the United States. Their TB burden is disproportionately greater after population adjustment, and their share of the national TB case count has increased, from 49.9% in 2012 to 51.3% in 2013. To continue to make significant progress toward TB elimination, TB control and prevention in the areas with the highest burden will have to continue to be given priority. One contributing factor to the geographic disparity is that these four states have populations at elevated risk for TB. In 2013, 16%–26% of the population in each of these four states was foreign-born (4). In addition, three of these states (California, New York, and Florida) were among the 15 states with the highest rates of homelessness in 2013 (5).

The rate of decline in TB incidence among foreign-born persons (2.1%) lagged behind the rate of decline among the U.S.-born (8.4%) in 2013, causing the proportion of TB cases in foreign-born persons to continue to increase. The majority of TB cases among foreign-born persons have been attributed to reactivation of TB infection acquired previously, with the rate reflecting TB incidence in their countries of origin (6). Further interventions aimed at diagnosing and treating latent TB infection (LTBI) among foreign-born persons are necessary to meet the goal of TB elimination in the United States.

What is already known on this topic?

Tuberculosis (TB) incidence has been declining in the United States since 1993, but an increasing proportion of cases have been among foreign-born persons.

What is added by this report?

For 2013, preliminary data show the number of TB cases reported in the United States was 9,588, an incidence of 3.0 cases per 100,000 population, compared with 3.2 cases per 100,000 population in 2012. Four states (California, Texas, New York, and Florida) reported more than half (51.3%) of all TB cases reported in 2013. Although TB cases among foreign-born persons in the United States continued to decline, the rate of decline in TB incidence since 2012 among foreign-born persons (2.1%) lagged behind the rate of decrease among the U.S.-born (8.4%), causing the proportion of TB cases in foreign-born persons to continue to increase.

What are the implications for public health practice?

Ongoing surveillance, vigilance, and prevention activities are needed despite the decline. Initiatives to improve awareness, testing, and treatment of TB disease as well as preventing TB by identifying and treating those with asymptomatic latent TB infection are needed to eliminate TB in the United States.

Persons experiencing homelessness also present a challenge for TB control. During 2006–2010, the TB rate among persons experiencing homelessness was estimated to be 36–47 per 100,000 population, approximately 10 times greater than the overall national TB incidence during that period (7). In addition, recent outbreaks among persons experiencing homelessness have underscored the potential for transmission in homeless shelters (8,9). Effectively addressing TB among persons experiencing homelessness requires partnerships between TB control programs and homeless service providers to diagnose and treat active TB disease and LTBI in this population.

The findings in this report are subject to at least two limitations. First, this analysis is limited to reporting provisional case counts and incidence rates for 2013. Second, incidence rates are calculated based on estimated population denominators from 2013. CDC’s annual TB surveillance report, which is released in September of every year, will provide final TB incidence rates based on updated denominators.

Although TB rates are declining in the United States, equal progress toward TB elimination is not being made in all populations. The disparity between TB rates in different populations defined by factors such as geography, country of birth, and housing status presents a challenge to TB control programs, given that strategies and interventions must be tailored to the population being served. Ongoing surveillance and an ability to translate surveillance data into public health action will be key to achieving TB elimination.

## Figures and Tables

**FIGURE 1:**
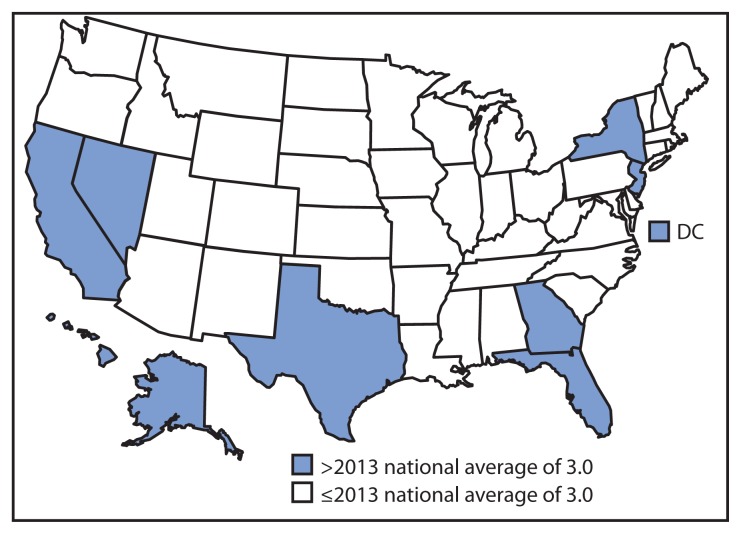
Rate^*^ of tuberculosis cases, by state/area — United States, 2013^†^ ^*^ Per 100,000 population. ^†^Data are provisional.

**FIGURE 2:**
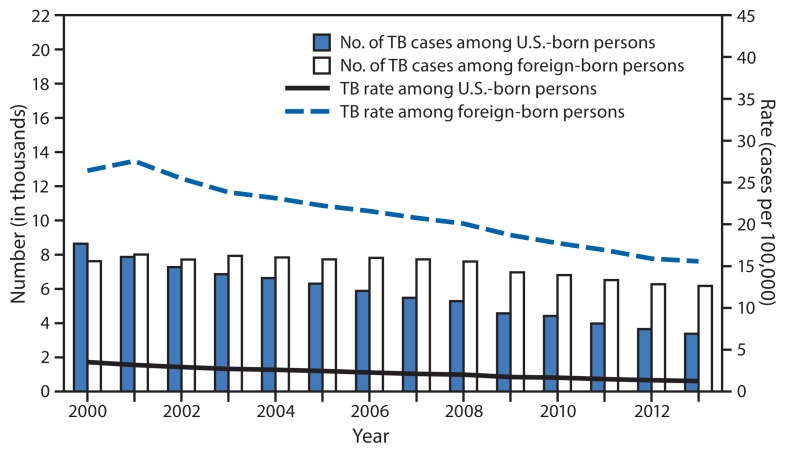
Number and rate^*^ of tuberculosis (TB) cases among U.S.-born and foreign-born persons, by year reported — United States, 2000–2013^†^ ^*^ Per 100,000 population. ^†^Data are updated as of February 24, 2014. Data for 2013 are provisional.

**TABLE:** Number and rate* of tuberculosis cases and percentage change, by race and ethnicity — United States, 2012 and 2013†

Race/Ethnicity	2012		2013		% change 2012 to 2013		Population	
			
	No.	Rate	No.	Rate	No.	Rate	2012	2013
Hispanic	2,790	5.3	2,698	5.0	(−3.3)	(−5.3)	53,027,708	54,165,861
Non-Hispanic								
Black	2,237	5.8	2,088	5.3	(−6.7)	(−7.5)	38,727,063	39,071,665
Asian	2,926	18.7	2,998	18.7	(2.5)	(−0.3)	15,619,997	16,050,150
White	1,571	0.8	1,427	0.7	(−9.2)	(−9.2)	197,705,655	197,823,217
Other§	388	4.4	334	3.7	(−13.9)	(−16.0)	8,833,617	9,048,925
Unknown	28		43					
**Total**	**9,940**	**3.2**	**9,588**	**3.0**	**(**−**3.5)**	**(**−**4.2)**	**313,914,040**	**316,159,818**

* Per 100,000 population.

† Data for 2013 are provisional.

§ Persons included in this category are American Indian/Alaska Native (2013, n = 125, rate = 5.4 per 100,000; 2012, n = 146, rate = 6.3 per 100,000); Native Hawaiian or other Pacific Islander (2013, n = 58, rate = 10.9 per 100,000; 2012, n = 63, rate = 12.1 per 100,000); and multiple race (2013, n = 151, rate = 2.4 per 100,000; 2012, n = 179, rate = 3.0 per 100,000).

## Footnotes

* Available at http://wwwn.cdc.gov/nndss/script/casedef.aspx?condyrid=876&datepub=1/1/2009%2012:00:00%20am.

† Additional information available at http://dataferrett.census.gov.

§ Includes Guam, the Commonwealth of the Northern Mariana Islands, American Samoa, the Federated States of Micronesia, Palau, and the Republic of the Marshall Islands.

¶ Defined by the World Health Organization as a case of TB in a person with a *Mycobacterium tuberculosis* isolate resistant to at least isoniazid and rifampin. Additional information available at http://whqlibdoc.who.int/publications/2010/9789241599191_eng.pdf.

** Defined by the World Health Organization as a case of TB in a person with an *M. tuberculosis* isolate with resistance to at least isoniazid and rifampin among first-line anti-TB drugs, resistance to any fluoroquinolone (e.g., ciprofloxacin or ofloxacin), and resistance to at least one second-line injectable drug (i.e., amikacin, capreomycin, or kanamycin). Additional information available at http://whqlibdoc.who.int/publications/2010/9789241599191_eng.pdf.
